# Patient and service-related barriers and facitators to the acceptance and use of interventions to promote communication in health and social care: a realist review

**DOI:** 10.1186/s12913-020-05366-4

**Published:** 2020-06-04

**Authors:** Gerard Leavey, Emma Curran, Deirdre Fullerton, Steven Todd, Sonja McIlfatrick, Vivien Coates, Max Watson, Aine Abbott, Dagmar Corry

**Affiliations:** 1 Bamford Centre for Mental Health and Wellbeing, School of Psychology, Ulster University, Coleraine Campus, Cromore Road, BT52 1 Coleraine, SA Northern Ireland; 2 Geriatrics, Altnagelvin Area Hospital (WHSCT) Glenshane Road, Londonderry, BT47 6SB Northern Ireland; 3 School of Nursing, Ulster University, Jordanstown Campus, Newtownabbey, BT37 0QB Northern Ireland; 4 School of Nursing, Ulster University, Coleraine Campus, Cromore Road, BT52 1SA Coleraine, Northern Ireland; 5 Adult Services, Northern Ireland Hospice, Whiteabbey Hospital, Doagh Road, Newtownabbey, BT37 9RH Northern Ireland

**Keywords:** Healthcare passport, Dementia, Realist review, Intervention, Design and evaluation [4–10]

## Abstract

**Background:**

More people living into old age with dementia. The complexity of treatment and care, particularly those with multiple health problems, can be experienced as disjointed. As part of an evaluation of a ‘healthcare passport’ for people living with dementia we undertook a realist review of communication tools within health and social care for people living with dementia.

**Aims:**

To explore how a ‘healthcare passport’ might work in the ‘real world’ of people living with dementia through a better understanding of the theoretical issues related to, and the contextual issues that facilitate, successful communication.

**Methods:**

A realist review was considered the most appropriate methodology to inform the further development and evaluation of the healthcare passport. We undertook a purposive literature search related to communication tools to identify (a) underlying programme theories; (b) published reports and papers on their use in various healthcare settings; (c) evidence on barriers and facliitators of their use.

**Results:**

Communication tools were noted as a way of improving communication and outcomes through: (1) improvement of service user autonomy; (2) strengthening the therapeutic alliance; and (3) building integrated care. However, while intuitively perceived to of benefit, evidence on their use is limited and key barriers to their acceptance and use include: (1) difficulties in clearly defining purpose, content, ownership and usage; (2) understanding the role of family caregivers; and (3) preparation among healthcare professionsals.

**Conclusion:**

Patient-held communication tools may be helpful to some people living with dementia but will require considerable preparation and engagement with key stakeholders.

## Background

The proportion of people living with dementia is rising [1, 2]. When dementia is accompanied by multiple and complex health problems, care and treatment can often be experienced as disjointed, with people being moved between services in a somewhat compartmentalised and fragmented process and with limited consultation with patient and their carers [3]. Thus, in the advanced stages of dementia, people may become doubly incontinent, unable to communicate their needs and at increased risk of hospitalisation, following chest and urinary tract infections and frequently experience pain, anxiety and swallowing problems.

The need for greater inter-sectoral and inter-professional care (e.g. between primary care, hospital and hospice) is amplified during episodes of illness and injuries, or where there is accelerated cognitive and functional challenges, adverse events and hospitalisation [4, 5]. Contact with multiple medical services reduces the likelihood of receiving holistic care and diminishes personhood [6] and may encourage disablement and marginalisation [7]. In this context, modern medical care is increasingly regarded as mechanistic and lacking compassion [8]. For example, a person living with dementia may have additional complex health problems such as diabetes and coronary heart disease, and/or may have physical disabilities. In Northern Ireland (NI) these diverse but often connected, problems are managed by different National Health Service (NHS) clinical services. Although health and social care in NI are ostemsibliy itegrated, socal care is sometimes outsourced to private or not-for-profit agencies, creating another layer of service contacts. There is no central coordination of specialist care. Consequently, patients and their families are often distressed by having to attend hospital outpatients on different days and, frequently, being obliged to explain their health problems, history and social contexts to a constantly changing cast of clnicians. The problem of fragmented care is not peculiar to the UK [8]. There is a need, therefore, for communication tools which indicate the needs and preferences of people with complex conditions and which obviate the difficulties of constantly restating health problems to health and socal care professionals. In response, people with chronic and life-limiting conditions, in collaboration with key stakeholder agencies, helped design a ‘healthcare passport’, maintained by the patient and containing a range of personal and medical information, updated by relevant health and social care professionals. As part of an evaluation of a communication tool (a “healthcare passport”) for people living with dementia [9], we undertook a realist review.

### Review purpose

To explore how a communication tool - a ‘healthcare passport’ - might assist people living with dementia to engage with health and social care providers [9]. To do this we examined the evidence of similar and relevant healthcare communication tools to facilitate a more in-depth understanding of potential implementation challenges [9].

## Methods

### Realist review

Realist methodologies increasingly inform the design and evaluation of complex interventions [10], unpacking the relationships between context, mechanism and outcomes of interventions and seeking to understand what works for whom, in what circumstances, and how [10]. Guidance on complex interventions [11] suggest that realist methodologies, can uncover the theories, often tacit, upon which programmes are based. Importantly, complex (active) programmes work only through the knowledge, beliefs, preferences and rationale (interpretation) of various stakeholders, for whom, different things may be at stake [10] . The complexity of dementia and dementia care (e.g. cognitive impairment, commonly occurring co-morbidity, loss of autonomy, and the prominent role of a caregiver proxy), we determined that a realist review was an appropriate method to uncover barriers and facilitators in the use of such tools, and provide insights and recommendations for the implementation of our own communication tool.

We adopted the Realist And MEta-narrative Evidence Syntheses: Evolving Standards.

(RAMESES) standards for realist syntheses frameworks [12] encompassing four interlinked phases including: 1. Programme theory development; 2. Evidence retrieval, data extraction and synthesis; 3. Programme theory testing and refinement through the evidence synthesis; and 4. Development of actionable recommendations.

### Programme theory development

We undertook an iterative process of consultation with stakeholder groups (service users, family care-givers and health and social care service providers, voluntary and statutory) and discussions within our interdisciplinary team which comprised three academic researchers (social science, social policy and psychology) and six health professionals from general practice, nursing, gerontology, palliative care and mental health. In addition, we held focus groups with service users and carers in order to explore the acceptability, content, and use of the passport. The in-depth discussions were carried out alongside a review of the literature. Following the consultation with stakeholders and the review of previous research on similar concepts, three theory areas were identified for greater exploration. These all related to communication approaches that were expected to enhance: 1) personhood; 2) integrated care; 3) therapeutic alliance and 4) self-management.

### Search process

We used a range of terms related to communication tools and ensured the inclusion of the most relevant material indexed in all major health, social and dementia welfare databases, as well as the grey literature. Keywords were identified through group discussion and previous systematic reviews. Specific ‘keywords’ varied depending on the database searched. With the support of a Health Sciences Librarian, one of the members carried out searches of six online databases.

The search strategies were kept as broad as possible (see Table 1). Some terms were combined and joined with the Boolean operator ‘OR’, to capture any instance of any term’s use. For example, “dementia” or “Alzheimer’s” or “Alzheimer’s Disease” and “patient-held records” or “family-held records”. Terms referring to communication strategies with the use of a passport were developed and incorporated into search strategies. Searches were completed in May 2016 to cover the years from 2005 to 2015. We also manually searched the publication references for papers included in systematic reviews. In keeping with the iterative, theory-building approach of realist reviews, we considered qualitative or discussion papers considered as informative to recommendations. In Phase 2, we included a process of evidence retrieval, data extraction and synthesis which involved searching for relevant evidence to test and refine the initial programme theory. In this phase, additional data extraction was carried out from the evidence and sources we identified as relevant. We focussed predominantly on the evidence base related to improving communication in dementia, but also explored the literature on communication interventions in areas (e.g. cancer, palliative care) with similar objectives and features. The consensus of the stakeholder groups was that while the passport should not be focussed on palliative care issues, evidence on advance directives or planning that might illuminate any shared issues related to the passport use. Thus, we also considered papers that addressed the facilitators and barriers associated with such communication tools. The search strategy located systematic and realist reviews, as well as primary studies.

**Table 1:** Databases and search terms

Databases searched	Search terms
Web of Science Medline CINAHL Embase PsycInfo Sociological Abstracts Grey literature Conference proceedings	healthcare; passport; handheld patient record; healthcare passport; patient-held records; family-held records advance directives; advance care planning; self-management; dementia; dementia care; Alzheimer’s Disease; AD Communication; communication tools Personhood; patient-activation; autonomy; therapeutic alliance; care triad; decision making and integrated care

A second team member reviewed the indexing terms and the databases searched, and team members reviewed the final abstracts and papers to ensure that the overall search strategy was executed appropriately.

### Inclusion and exclusion criteria

**Fig. 1:**
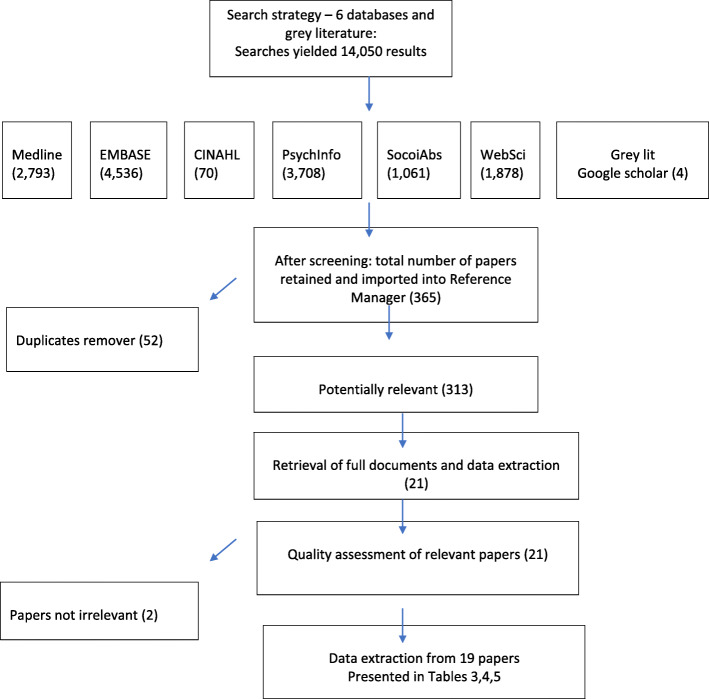
Search strategy and data extraction

We included reports of dementia specific workforce, practice and/or organisational development programmes and interventions (combinations of these may vary). Also, included is evidence supporting or rejecting the use of patient-held communication tools in other chronic and life-limiting conditions. Perhaps unique to realist reviews, we did not exclude evidence unless it was unrelated to the identified theories (Fig. 1).

## Results

For the purpose of this realist review we have conceptualised the theoretical construction underlying patient patient-held communication tools. Second, the synthesis of evidence was intended to inform and clarify our understanding about conceptual, instrumental and the direct impact of communication tools.

### Theoretical construction of the ‘healthcare passport’

Three overarching theoretical frameworks related to patient-held records or health documents were noted as a way of improving communication and outcomes. First, service user autonomy may be improved; (2) the therapeutic alliance can be strengthened; and (3) integrated care can be achieved, facilitating inter-agency working. Incorporating these concepts as underlying frameworks should affect the three levels of action [10] as prerequisites for successful implementation of a complex intervention. These levels are: (a) at the service user level; (b) at an intrapersonal level and relations between the patient, their informal carers, and clinicians (i.e. *the care triad*): and (c) patient activation may be promoted at an interpersonal level and at the level where care is organised. Through cross-referencing these three frameworks we highlight the levels of action which may support the ‘healthcare passport’ and the anticipated outcomes.

### Enhancing user autonomy and self-management skills

The initial framework underlying the ‘healthcare passport’ is the enhancement of the user’s autonomy [13, 14]. This benefits the person with dementia at an intra-personal level by encouraging active involvement in treatment [14–16], assisting understanding any symptoms and warning signs of deterioration. Importantly, service users feel better supported at both social and organisational levels [17, 18]. Enhanced service user autonomy through improved communication assumes that empowerment is achieved and equal power dynamics will evolve [19, 20] [21]. Moreover, service user autonomy links to the theory of patient activation [22] defined as ‘an individual’s knowledge, skill, and confidence for managing their health and health care’ [22], increasingly important in the context of co-morbidity [23].

Self-efficacy theory, emphasising the importance of an individual’s confidence in accomplishing specific goals [24] [25], is a behaviour-specific psychological attribute which can be learnt or enhanced [25] and may be predicated on individualised coping strategies in dealing with varying task-orientated demands and situational environments [26] [27] [28].

### 2nd framework: strengthening the therapeutic alliance

We anticipated that the use of the healthcare passport would strengthen the therapeutic alliance [29] through information exchange between service users and clinical staff [20] which, in turn may increase mutual understanding and treatment compliance [30]. In addition, stakeholders’ information needs might be facilitated and shared decision-making may also be promoted [13, 16, 31]. Consequently, quality of life and quality of care may be improved [32]. At the organisational level, early identification of healthcare problems and timely intervention may be easier to achieve through improved communication, providing that the patient and/or informal carer has been provided with appropriate information. In this way hospital admission may be avoided. However, implicit in the design of the passport is that a meaningful partnership in dementia care is achievable through promotion of person-centred care rather than through the more established medical model, the focus of which is on the treatment of individual disease or symptoms decontextualized from social circumstances and environment [7, 33].

### 3rd framework: integrated care

The third framework is based on integrated care, the ‘joined-up’ partnership of health care providers. While modern health care systems have invested in electronic sharing of information between health care professionals, they remain limited in their reach. They also exclude the patient and carer from this information exchange. Moreover, while complex, multimorbid conditions and health care contacts require information-exchange and advice to the patient and family caregivers, there are no single platforms to permit this.

The ‘healthcare passport’ is intended as a tool to assist the co-ordination of care between various health and social care professionals by which the various care needs of the user can be documented and gaps in care avoided [19, 34] [35]. The success of the ‘healthcare passport’ depends on practitioners’ willingness to share information and knowledge, but also requires regular updating when conditions and circumstance change. Clinicians may regard this a burden. There is also an assumption of ‘openness’ in the entries made by professionals (and by carers) but this may not be the case as it may sometimes involve ethical consideration and degrees of self-censorship as to what might be helpful or injurious.

### Evidence retrieval, data extraction and evidence synthesis

Initial searches provided 14,050 papers. After screening, we imported 362 papers into a database; following removal of duplicates and screening for quality, we identified 310 potentially relevant papers were located. After initial screening, twenty-one papers were quality assessed, data extracted and synthesised in a narrative format. Two of the the 21 papers were deemed by the research team to be irrelevant and were subsequently removed. The final selection comprised thirteen were reviews (systematic or realist reviews), one was an opinion paper, and six reported on evaluations of communication tools for different patient groups. Three of the primary studies described evaluations of tools for people with dementia. Tables 2 and 3 provide a summary of the included reviews, and Table 4 provides details of the primary studies.

**Table 2:** Summary of reviews focused on approaches to improve communication

Author & year	Focus of review	Number of studies included or type of study	Summary of key findings
Campbell et al. (2009) [36]	**Cochrane review:** examined advance statement leads to less hospitalisation (either voluntary or involuntary). **Population** People with mental illness **Interventions** Advanced directives	Search strategy described Inclusion criteria: RCTs The review located 2 trials of *n* = 321 patients	**Findings:** Limited evidence to make definitive recommendations. More intensive forms of advance directives show promise, but currently practice must be guided by evidence other than that derived from RCTs. More trials are indicated to determine whether higher intensity interventions, such as joint crisis planning, have an effect on outcomes of clinical relevance.
Gysels, et al. (2006) UK	**Review:** patient-held record (PHR) in cancer care **Population** People with cancer **Intervention** Patient held records (PHR)	Systematic Review Seven RCTs and six non-experimental studies were identified.	**Findings:** • randomized trials found an absence of effect • non-experimental evaluations shed light on the conditions for its successful use. Most patients welcomed the introduction of a PHR. Main problems related to its suitability for different patient groups and the lack of agreement between patients and health professionals regarding its function. • Further research is required to determine the conditions under which the PHR can realise its potential as a tool to promote continuity of care and patient participation.
Hayhoe, et al. (2011) UK [37]	**Narrative Review** of Advance Care Planning (ACP) based on a narrative review of the literature **Population** Not specified I**ntervention** Advance Care Planning (ACP)	Narrative review (no inclusion/exclusion criteria) PubMed was searched using the terms ‘advance care planning’, ‘living wills’, ‘advance decisions’.	**Findings:** ACP is supported by both patients and doctors and has documented benefits in extending autonomy and facilitating decision-making. Though currently under-used, partly through widespread lack of knowledge, it could be a valuable asset in end of life care if routinely discussed with patients. May be relevant for older people, at greater risk of conditions affecting their capacity to make decisions. Problems include the anticipation of potential future clinical scenarios, the complex assessment of capacity to participate, and concerns about coercion. Additionally, ACP create a sense of urgency in ensuring that discussions take place while patients still retain capacity. Continued low uptake of ACP suggests that further education of both public and health-care professionals need to take place regarding the benefits of ACP.
Kawi (2012) Review USA [38]	**Concept analysis** of self-management support (SMS) to provide clarity for systematic implementation in practice. **Population** Chronic conditions **Intervention/approach** Self-management support (SMS)	Concept analysis – narrative review No inclusion/exclusion criteria Rodgers’ evolutionary concept analysis method was used. Data sources included systematic multidisciplinary	**Background:** SMS refers to comprehensive sustaining approaches toward improving chronic illness outcomes consisting of patient-centred attributes (involving patients as partners; providing diverse, innovative educational modalities specific to patients’ needs; individualizing patient care), provider attributes (possessing adequate knowledge, skills, attitudes in providing care), and organizational attributes (putting an organized system of care in place, having multidisciplinary team approach, using tangible and social support). SMS is a concept in its early phase of development. It is increasingly evident in literature on chronic illness care. However, the definition has been simplified or vague leading to variable SMS programs and inconsistent outcomes. Elucidation of SMS is necessary in chronic illness care to facilitate clear understanding and implementation.
		searches of multiple search engines.	**Implications:** A well-clarified SMS concept is important in theory development. The attributes offer necessary components in SMS programs for systematic implementation, evaluation, and research. There is great potential that SMS can help improve outcomes of chronic illness care.
Ko et al. (2010) Australia [39]	**Review**: patient-held medical record (PHR), compared to usual care, improves clinical care, patient outcomes or satisfaction. **Population** Chronic conditions **Intervention/approach** Patient Held Records (PHR)	14 studies, explored the use of PHR in diabetes, oncology, mental health, rheumatoid arthritis, stroke and palliative care.	**Findings:** The studies used a variety of designs of PHR and compared this with usual care. PHR were implemented with varying degrees of patient and staff support and education, mainly for six months or less. Outcomes included attitudes on the usefulness of PHR, the quality of information exchange, process indicators, and clinical and physiological indicators. The evidence on the effectiveness of PHRs is generally of low or very low quality. These studies do not demonstrate a significant benefit of introducing PHR. **Conclusions:** There is no clear benefit of implementing a PHR, and due to medium to high risk of bias these findings should be interpreted with caution. **More high-quality studies are needed to evaluate properly the effectiveness of PHRs in chronic disease populations.**
McCorkle et al. (2011) USA [40]	**Review:** self-management interventions. **Population** Patients with cancer **Intervention/approach** Self-management	RCTs (*n* = 32) of self- management interventions.	**Findings:** Self-management is poorly defined and a common set of self-management actions for cancer care notwithstanding, oncology practices can now build strong relationships with their patients and formulate mutually agreed upon care plans that enable and empower patients to care for themselves in the way they prefer.
Murphy et al. (2016) Ireland	**Cochrane Review:** improving palliative care delivered to people with advanced dementia **Population** Patients with advanced dementia **Intervention** Interventions aimed at improving palliative care	Cochrane Review Extensive searches and inclusion criteria described 2 studies located both from the USA	**Findings:** This review focused on interventions aimed to impact one or more of the following domains: • The PLWD focusing on managing pain or on psychological, social, or spiritual dimensions of the patient • The family/carer, with an emphasis on carer well-being, carer burden and grief or bereavement support • The quality of care, which may include interventions such as advance care planning, staff education programmes or the organisation and delivery of care. **Conclusions:** Giving relatives this information made it a little easier for relatives to make decisions about what methods would be used to feed the person with dementia. Very little high-quality work has been completed exploring palliative care interventions in advanced dementia
Nicaise, et al. (2013) [41]	**Realist systematic review** of Psychiatric Advance Directives (PADs); (e.g. Treatment preferences during crisis) **Population** People with severe and chronic mental illness **Interventions** Psychiatric advance directives	Forty-seven studies were retrieved, ranging from 1996 to 2009.	emergency care, or reduction in the resort to coercion. T**he shape of the whole intervention at each stage relies on such clarification. More research is needed, particularly on the later stages of the intervention, as the evidence for how PADs should be implemented is still incomplete.**
Reilly et al. (2015) Cochrane Review UK [42]	**Cochrane Review:** case management approaches to home support for people with dementia. **Population** PLWD **Intervention/approach** Case management	13 RCTs involving 9615 participants with dementia in the review.	**Findings:** Some studies examined the benefit of case management in reducing admissions to residential or nursing homes (institutionalisation). They found benefits at six months and 18 months but not at 12 and 24 months. Case management increases the use of community services but there was some indication that overall healthcare costs may be reduced in the first year. Some studies reported that case management was no more effective than usual care in improving patient depression, functional abilities or cognition. There was not enough evidence to clearly assess whether case management could increase the length of time until people with dementia were admitted to care homes.

**Table 3:** Summary of reviews focused on needs of PLWD

Author & year	Focus of review	Number of studies included or type of study	Summary of key findings
Dooley, et al. (2015) Review UK	**Review:** observational studies of communication between patients, companions and healthcare professionals **Interventions** None**.**	Eight databases searched. 23 studies were identified observing: diagnostic, follow up, day centre, primary care and research consent interactions. Companions were present in 14 studies	**Findings:** Three themes emerged: emotional impact of diagnosis, level of patient involvement and participant strategies to save face and cope with cognitive impairment. Varying patient involvement, showing marginalization in primary care but not in assessments or diagnostic feedback. Patients used humour and metaphor to compensate for difficulties retrieving information and responding appropriately, suggesting preserved awareness of the pragmatics of interaction. Companion roles fluctuated between patient advocate and professional informant. Professionals encountered challenges adapting to heterogeneous patient groups with varying capabilities and needs. Patient-companion professional communication in dementia care raises various ethical questions: Healthcare professionals need guidance in delivering a diagnosis and strategies to optimize patient and companion participation.
Street (2013) Essay/Opinion paper USA	**Theoretical paper:** clinician– patient communication contributes to a patient’s health and offers recommendations for future research.	No search strategy described.	**Findings:** communication measurement is complicated because relationships among communication behaviour, meaning, and evaluation are complex. **Conclusion:** Researchers must do more to model pathways linking clinician–patient communication to the outcomes of interest, particularly pathways in which the communication effects are indirect or mediated through other variables. To better explicate how communication contributes to health outcomes, researchers must critically reflect on the assumptions they make about communication process and choose measures consistent with those assumptions.
Van Der Roest et al. (2007) Qualitative review The Netherlands	**Narrative review** on the subjective needs of people with dementia **Population** PLWD living in the community **Intervention:** none.	Narrative review 34 studies	**Findings:** Few studies specifically aimed to measure the needs of people with dementia. Reported most frequently by people with dementia was the need to be accepted and respected as they are, the need to find adequate strategies to cope with disabilities, and the need to come to terms with their situation. Overall, people with dementia do not frequently mention how they want their needs to be met.
Steeman et al. (2006) Belgium [43]	**Narrative Review:** qualitative studies on the management of daily life with dementia. **Intervention:** None	28 qualitative studies (reported in 33 articles)	**Findings:** Memory loss threatens perceptions of security, autonomy and being a meaningful member of society. Individuals use self-protecting and self-adjusting strategies to deal with perceived changes and threats. Thereby causing frustration, uncertainty and fear. Results support the integration of proactive care into the diagnostic process, to improve quality of life. **Care should actively involve both the individual with dementia and their family.**
Barlow et al. (2002) [44]	**Review:** self-management approaches for people with chronic conditions	145 papers on chronic conditions	**Findings:** No studies of self-management found for dementia (most chronic conditions covered were asthma, diabetes, arthritis). Self-management approaches mostly group-based, individualised, or a combination of both. Group approaches were often supplemented with written materials and audiotapes. The format of self-management approaches varied and included booklets, lectures, role play and goal setting. Most approaches combined at least two formats of delivery (e.g. lectures and manual). Evidence from RCTs suggests that self-management approaches may be effective in increasing participants’ knowledge, symptom management, use of self-management behaviours, self-efficacy, and aspects of health status (e.g. depression). However, not all approaches target all of these outcomes. Multi-component programmes do not show improvements on all outcomes.

**Table 4:** Primary studies of evaluations of interventions to improve communication

Barber, et al. (2015) Evaluation UK	**Aims:** An evaluation of ‘My Medication Passport’ - its value to **older patients** **Population**: Older patients I**ntervention**: A booklet, recording details on patient’s medicines.	A total of *N* = 200 patients were given the passport Follow-up study of *n* = 133 who participated in structure telephone interview interviewed 40% aged 70+ years	**Findings:** More than half of the respondents had found their medication passport useful or helpful in some way; 42% through sharing details from it with others (most frequently family, carer or doctor) or using it as a platform for conversations with healthcare professionals.; One-third of those questioned carried the passport with them at all times. Conclusions: My Medication Passport has been positively evaluated; the study provided a better understanding of (a) how it is used by patients, (b) what they are recording and (c) how it can be an aid to dialogue about medicines with family, carers and healthcare professionals. Further development and spread is underway including an App for smartphones that will be subject to wider evaluation to include feedback from clinicians.
Ito, et al. (2015) Japan Evaluation [45]	**Aims:** utility of patient-held records **for patients with dementia** in the community. **Population** PLWD living in community **Intervention** patient-held records	workshops with health professional across Japan on Family held/ patient-held records. Searches of the literature were also conducted.	**Findings:** The searches identified were eight sets of family-held/patient-held records in Japanese communities of various sizes, all of which were aimed at integrating information from various services, including information provided by medical and psychiatric professionals to the family and patient. The review did not examine effectiveness of these tools. Innovative tools have been available in the areas of the hopes and preferences of the patient, medication and monitoring, sharing information, and the use of information technology. Family held/ -held records may have potential as a tool to enhance the integrated care of people with dementia.
Stacy, et al. (2008) USA [46]	To examine (a) **mothers**’ satisfaction with use of a personal parent-held child health record (PHCHR), (b) frequency of use (c) behaviour changes, and (d) perceived barriers **Intervention** Patient held records	A total of *N* = 100 mothers were given the PHCHR for one year. *n* = 82 mothers completed the 22-item validated evaluation instrument.	**Findings:** Patients reported high levels of satisfaction with all applicable use of the PHCHR. Respondents believed the PHCHR was a useful tool that served as a cue to increase their action in health seeking behaviours.
Robinson et al. (2010)	An intervention to improve patient centred communication in outpatient reviews of **patients with dementia**. A thematic analysis of recordings, with interviews and literature review. **Population** **PLWD** **Intervention:** **No specific intervention – exploratory study**	Semi-structured interviews with patients, carers and clinicians on their views about barriers and facilitators to patient centred care	Difficulties included: • Developing a therapeutic alliance, especially with patient companion conflict • Facilitating shared responsibility whilst promoting patient autonomy • Presenting information in manageable amounts so that patients with dementia can make informed decisions • Exploring person with dementia’s experience and promoting quality of life. • People with dementia very rarely identified issues in response to direct questions • Consultations tended to focus on negative aspects of a patient’s life. • The way in which doctors structured their consultations could be as important as the communication skills they use.
Young, et al. (2011) UK	Development of a communications advice package for PLWD. **Population** PLWD I**ntervention** Communication Advice Package (for professionals)	Iterative consultation process with multidisciplinary professional and lay stakeholders, including PLWD in the UK. Stakeholders were asked to reflect in detail on their own experiences of communication in relation to dementia.	**Findings:** Participants reported dissatisfaction with current communicative practices, particularly during contact with medical professionals. Both lay and professional participants reported general dissatisfaction with currently available communication advice. An agreed version of a dementia toolkit for effective communication (DEMTEC) was produced. This consists of three “levels”. • The foundation Level details beliefs about the psychosocial effects of dementia on communication, as well as empowering approaches to communication involving PLWD. • Level 2 consists of practical considerations and advice in eight key areas. • Level 3 uses case studies to show how the principles and advice in preceding levels are applicable to individuals in different care contexts and at different stages of dementia. This project has produced a free-to-users instrument that is empirically supported and adaptable.

Fourteen of the papers described approaches or interventions aimed at improving communication. Two reviews [39, 47] and three primary studies [45, 46, 48] described the evidence on the effectiveness of patient held records with a range of different populations including PLWD, mothers of young children, people with chronic conditions. Two reviews [36, 41] examined the research evidence on the advance directives for people with mental illness, and one primary study used an RCT to evaluate the effectiveness of Joint Crisis Planning (JCP). Advanced care planning was examined in a narrative review by Hayhoe et al. [37]. Approaches to improve self-management were the subject of a concept analysis and a review [38, 40]. Two reviews [42, 49] explored the literature on approaches to improve more integrated care. Table 5 presents a description of some of the approaches described in the reviews and primary studies.

**Table 5:** Description of some of the approaches described in the located literature

Term	Description
Advanced Treatment Directives (ATD)	Advance Treatment Directive (ATD) is a document that specifies someone’s future preferences for treatment, in the event that they lose the mental ability to make treatment decisions (i.e., lose capacity). They have originally been used to instruct treatment for end-of-life, but since people with mental health difficulties may also have periods where they are unable to make treatment decisions, an advance statement could assist with selecting appropriate medication, specifying wishes regarding child care, and choices in a number of other areas of their life and treatment.
Joint Crisis Plan (JCP)	The Joint Crisis Plan is a patient’s negotiated declaration regarding treatment preferences for any future psychiatric emergency when they might not be able to express their wishes.
Patient-Held Records (PHR)	Patient-Held Records (PHR) are tools intended to inform and involve patients in their care, and to assist communication between the various groups of people who are professionally and informally caring for the patient, thus facilitating continuity of care. They are described as ‘logbooks’, ‘patient travelling records’, ‘personal records’, ‘client records’, ‘shared care records’, or ‘care diaries’. They can take numerous forms ranging from a dynamic tool used by the patient and all health-care professionals providing care to the patient, to a print-out from the patient’s medical record, or general information sheets.
Psychiatric Advance Directives (PAD)	Psychiatric Advance Directives (PADs) are documents that allow individuals with severe and chronic mental illnesses to determine their treatment preferences for future crises and to assign a proxy decision-maker for any periods of incompetence.
Self- Management Support (SMS)	Self-Management Support (SMS) refers to wide-ranging sustaining methods for improving chronic illness outcomes, based on patient-centred attributes (such as involving patients as partners; providing varied, advanced educational methods specific to patients’ needs; and individualising patient care), provider attributes (such as possessing suitable knowledge, skills, and attitudes for care provision), and organisational attributes (such as putting an organised system of care in place, having a multi-disciplinary team approach, and using tangible social support).

### Evidence synthesis

Shared information and standardised systems between different providers and clinicians, even within the same organisation, are rare or sub-optimally provided. Beyond advanced care directives or planning in palliative care, there are few communication tools such as the ‘healthcare passport’ for people living with dementia. In other clinical areas where patient held records have been used, the evidence for effectiveness is somewhat limited. Often, it is not a question of the acceptability of PHR but rather, differences between patients and health professionals about their function. In palliative care, the provision and uptake of advance care planning remains limited, stuck as it seems, between widespread theoretical acknowledgement and a pervasive reluctance to use it on religious-ethical (Cultural) grounds. Again, in line with other findings we noted a general failure within dementia studies to apprehend the complexities of living with dementia, and thus, the need for multifactorial interventions to assist communication and effective care [50].

### Diagnosis and support

Healthcare services should continue to address any concerns of people who have been diagnosed with dementia and provide information about symptoms that might be experienced. Literature in this area has identified several common needs that a person with dementia might experience soon after their diagnosis: the need for an explanation; the need to relieve the pressure of maintaining a normal appearance; and the need to feel supported [43]. Unfortunately, the evidence on effective psychological self-management support components is of limited quality [51]. However, minimally, it may be helpful for service providers to develop written care plans or provide a document to convey important information at the time of diagnosis and contact with services [51]. Patients reported being most likely to share such a document with members of their family and many commented on how useful it was to both patients and carers.

### Case-management

People living with dementia in the UK have typically 4.6 additional long-term medical conditions. Yet, health-care services are ordinarily structured with a focus on single conditions, rather than taking a whole-person approach. Health care policy in the UK has recommended the introduction of a comprehensive system for case management [52] on a systemic level. Emerging GP commissioning consortia concentrate on authorising more cost-effective models of care for people living with dementia [53], and co-ordinating effective care has been highlighted in recent guidance [54]. A current report has indicated that case management could significantly reduce health-, and social care costs, though the costs of unpaid care are likely to rise regardless [55].

Case management interventions could overcome the difficulties with service fragmentation and ease the burden on carers. However, definitive evidence for case management is elusive given the heterogeneity of diseases, interventions, outcome measures and reporting style [42]. Issues of substitution, and auxiliary information were not always presented in the studies [39]. Therefore, a recommendation for future studies is to demonstrate the extent to which case management interventions were delivered as planned. It is advisable to have well-developed training and protocol manuals to ensure the fidelity and replicability of the intervention [35].

### Patient held records

A review of patient-held records [35] identified 14 eligible studies which included diabetes, oncology, mental health, rheumatoid arthritis, stroke, and palliative care. Bias was notable in most of these, with little evidence that PHRs in these areas are effective. Patients and staff reported no noticeable benefits from the intervention, compared with normal care. Furthermore, in two of the studies staff expressed resistance to using the PHR as a result of an already heavy work load [56, 57]. The contents were often unclear, or combined the PHR with supporting components (e.g., education, intervention coordinators, and/or posters in clinics), and some studies provided a PHR with minimal written instructions on its use. Consequently, it is problematic to identify a ‘standard’ PHR, even with specific disease areas. Furthermore, the duration of implementation, which was mostly limited to between three and 6 months, is, in many cases, likely to have been inadequate.

To aid the implementation of a PHR, the following changes are worth considering: the form and content of the PHR; attitudes of staff to using the PHR; and attitudes of patients to being proactive in their own care. To increase the success of a PHR intervention, future studies might consider the best application of supporting tools to increase the effectiveness of a PHR. Additional organisational support, such as coordinators, may have mitigated this barrier to implementation in some studies, but are likely to be unavailable in most clinical environments. Staff ‘buy-in’ and managerial support could impact the outcomes of PHR interventions, but these are not sufficiently described. Notably, most of the reviewed studies showed that patient commitment to the PHR was low.

Formerly, effective use of the PHR was compromised by the low level of engagement from health professionals. It was often not applied by health professionals as intended, i.e., for the sharing of care with patients. Instead, they tended to use it as a means of communication with other professionals rather than with patients. It was found that the PHR served separate functions for health professionals who treated it as a document to confer technical information, and for patients for whom the record represented a means through which to express their values, and views about treatment and future care. In all studies, the PHR was meant to operate both as a clinical and as an informal document, yet the importance of the latter in the management of a patient’s care was sometimes overlooked. Despite its patient-driven purpose, health professionals have a key role to play in the acceptance and successful application of the PHR.

We found no publications relating to an evaluation of patient-held (or family-held) records for dementia. However, a review of family-held/patient-held records that were developed in collaboration with psychiatric services patients was undertaken [58] in Japan, where dementia is managed through psychiatric services. The researchers did not evaluate the PHRs but purely sought to report their existence, aims, and content. The aim was to reduce fragmentation-, and improve continuity and coordination of health care.

In an evaluation of a medication passport (MP) [48] most patients reported positive results; feeling that their MP was useful, that it facilitated dialogue about medicines, and that this patient-held portable document was appraised by its users as ‘a good idea’. Most patients reported that the passport facilitated communication between them and their health professionals, and carers regarded it as a helpful reference point when communicating with the patient or healthcare professionals [48]. Nevertheless, less than a fifth discussed their passport with their GP, while ownership and application of the passport were unclear.

### Advance directives

As noted earlier, guided by concerns for patient needs and preferences for care, the healthcare passport was originally conceptualised with advance directives in mind. A realist systematic review which investigated the use of Psychiatric Advance Directives (PAD) noted various challenges of a complex and multistage intervention [56, 57]. Although the document was initially intended to promote the patient’s autonomy, findings clearly indicated that PADs are more effective in improving the therapeutic alliance. In support of this it has been reported that when enablement features are designed to assist users in completing a PAD or JCP, are practical and responsive to users’ interests and needs, it will increase the rates of acceptance and improve the working alliance [59]. Decision making becomes even more difficult when individuals lose the capacity to express their wishes, and in these circumstances health professionals rely on information from others in order to make decisions based on their patients’ best interests. Advance care planning should help with making these choices clearer, based on the documented preferences of what the patient would have wanted were their capacity still present. However, such documents are rarely used yet, and where they are, health-care professionals are often cautious due to the potential multitude of ethical and legal problems.

Authors of an RCT focused on joint crisis plans (JCP) concluded that the best measured outcomes were obtained when the designation of the document and its content were discussed among all of those in the care triad [41]. Although the primary use of a PAD intends to enhance the users’ autonomy, professional ‘buy in’ is key to execution. PAD endorsement is higher when professionals play a key role in the development of the document, making them less inclined to override the directives [60]. The designation of a surrogate decision-maker may assist in the uptake of PAD. However, in some instances, having this designation may unintentionally produce relationship conflict and so weaken the therapeutic alliance.

An operational barrier to the use of PADs is that they have a range of different expectations placed upon them by various stakeholders, most obviously and challengingly the conflicting demands from clinicians and patients, obscuring the purpose and possibly responsible for the low acceptance rate [61]. These expectations need to be clarified at each stage to ensure a successful intervention.

Thus, PADs understood in terms of therapeutic alliance are more feasible than the ‘classic PAD’. There are concerns about prescriptive PADs that service users may refuse treatments, and potential conflicts resulting from surrogate decision-making, although these concerns can be addressed when a PAD is completed in consultation with health professionals within a therapeutic alliance framework [59].

A review of 145 self-management interventions for people with chronic conditions [62] did not note any studies in dementia, indicating that the progressive nature of the condition may provoke the perspective that self-management has no relevance. Research in this area is still rare and mostly limited to qualitative design, concept development, and pilot studies for people living with early stage dementia [44, 63]. The synthesis of the evidence on interventions supporting self-management for people with dementia indicates that healthcare services need to provide information about symptoms, and about associated symptoms. This material should also be provided to family and other informal carers [51]. The ‘stigma’ surrounding dementia and its progressive nature [43] can make discussion about it covert and difficult. The literature identifies a number of shared needs at the time close to a diagnosis of dementia, namely the need for an explanation, the need to relieve the pressure of maintaining a “normal” appearance, and the need to feel supported [59].

Parent-Held Child Health Records (PHCHR) offered to pregnant women and mothers of young children have been used widely for some time. Although employed in a very different area, we wished to examine the factors that made them acceptable and popular over many years [64–68]. Behaviour changes associated with the use of the PHCHR included improvements in: knowing when to call the doctor; keeping appointments; maintaining vaccination schedules; obtaining prenatal care; and staying informed about their own and their child’s/children’s health. Low uptake was not a concern in this population but the reason provided most frequently for not using the PHCHR was low engagement from staff members. This suggests that the PHCR is held by parents with a clear, central aim of building and safeguarding their child’s health at its most vulnerable stage. It is thus, highly focussed and structured towards appointments and key milestones, rather than dealing with multiple concerns and pathology.

## Conclusion

Optimally, health professionals should communicate effectively with patients, patient’s families, peers and colleagues consistently throughout the individual’s care [69]. Healthcare passports may be helpful to some people living with dementia but as this realist review has demonstrated there are few interventions that attempt to minimise the challenges of fragmented care and poor communication for this population. Moreover, future interventions of this kind must anticipate that access and usage are likely to be affected by multiple and complex factors, including, differential and conflicting stakeholder needs and expectations, professional buy-in, uncertainty of passport aims and functions and the mundane human complications of passivity and forgetfulness. Attention should be paid to the importance of these relationships at the different stages of the disease progression in dementia. Thus, while the individual’s condition may deteriorate and their needs may become more complex, it should be acknowledged that their environment and social circumstances and support is also likely to undergo change. A patient’s needs may be attended to within a delicate ecosystem of various carers, each one reliant on each other and the stability of certain factors and conditions, social and interpersonal; when any one of these factors is disturbed or compromised there are likely to be ramifications for the provision care.

### Summary of actionable recommendations

1. Healthcare passports may be directed for the ultimate benefit of the patient but it should be acknowledged that they will also benefit family members and healthcare professionals.
2. Healthcare passports can only meet their intended utility in a system of relationships. These relationships (stakeholders) should be identified prior to the inception of the passport.
3. Multiple and varying expectations makes the purpose of passport-type interventions unclear and may explain the low take-up rate. Future development of healthcare passports may require a more focussed consideration of their aims, and thus, content.
4. Acceptance of and commitment to the passport from primary care, community and hospital staff is essential. GPs may be particularly pivotal in the success of passports but may be reticent to be involved. This will require high level policy engagement, managerial commitment and additional resources for discussion, consultation and training. HCPs need to be persuaded that the benefits of the passport outweigh the perceived additional staff burden. More generally, the evidence suggests that staff at all levels, including more senior staff, need appropriate training on dementia.
5. A patient’s diagnosis of dementia is often not shared with other health professionals and thus may compromise effective care and treatment. A diagnosis of dementia should be flagged up on medical/electronic records. This should include systems for automatic updates of a dementia diagnosis to be transferred to other health-care services that the PLWD.
6. To improve the implementation of healthcare passports (acceptability and usage) there is a need for auxiliary tools such as manuals (e.g. CD-Rom or internet-based) and other explanatory materials. These may require evaluation. Well-developed manuals and protocols should be more widespread, since they can help to ensure the transparency, replicability and integrity of a complex intervention.
7. The ownership and shared use of healthcare passports need to be clarified. To do this, it should be acknowledged that the circumstances, capacities, needs and support, among other things, of individuals are different for each service user and that these factors may also change over time.
8. Families are crucial to the care of people with dementia, especially as the person gets older and the disease progresses. Their role as proxy decision-maker of the passport is likely to require additional discussion and agreement. Goal-setting and environmental adjustments including carer training in problem-solving strategies were also found to be important components of family carer interventions.
9. The use of models such as the triangle of care model may be helpful in ensuring that the input of family carers is properly recognised. This should include appropriate training in carer engagement for staff, and policy and practice protocols regarding confidentiality and information sharing.

## Data Availability

Not applicable.

## Abbreviations

AD: Alzheimer’s disease
AS: Alzheimer’s society
ATD: Advanced treatment directive
EOAD: Early onset alzheimer’s disease
EPO: Engagement and participation officer
GP: General practitioner
HCP: Health care professional
HP: Healthcare passport
HSCP: Health and social care professional
JCP: Joint crisis plan
LD: Lewybody dementia
MP: Medication passport
OT: Occupational therapist
PADs: Psychiatric advance directives
PCC: Person centred care
PD: Parkinson’s disease
PHA: Public health agency
PHCHR: Parent-held child health record
PHR: Patient-held records
PLWD: People living with dementia
QLR: Qualitative longitudinal research
QoC: Quality of care
QoL: Quality of life
RCGP: Royal college of general practitioners
RCT: Randomised controlled trial
SMS: Self-management support
WHSCT: Western health and social care trust
